# Supplementary material for: Resolving the full spectrum of human genome variation using Linked-Reads
Source: Genome Res. 2019 Apr;29(4):635–45. doi: 10.1101/gr.234443.118 (PMC6442396; doi:10.1101/gr.234443.118)
Supplement: Supplemental Material [file supp_gr.234443.118_Supplemental_longranger-2.2.zip › longranger-2.2.tar.bz2!/lr2.2-public/lib/stan/cmdstan/stan/doc/api/html/matrix_2promote__scalar_8hpp_source.html]

Stan: src/stan/math/matrix/promote\_scalar.hpp Source File


|  |  |
| --- | --- |
|  | Stan  2.5.0  probability, sampling & optimization |


- Main Page
- Related Pages
- Namespaces
- Classes
- Files

- File List
- File Members

All Classes Namespaces Files Functions Variables Typedefs Enumerations Enumerator Friends Macros Pages

- src
- stan
- math
- matrix

promote\_scalar.hpp

Go to the documentation of this file.

1 #ifndef STAN\_\_MATH\_\_MATRIX\_\_PROMOTE\_SCALAR\_HPP

2 #define STAN\_\_MATH\_\_MATRIX\_\_PROMOTE\_SCALAR\_HPP

3

4 #include <stan/math/functions/promote\_scalar.hpp>

5 #include <stan/math/matrix/promote\_scalar\_type.hpp>

6 #include <stan/math/matrix/Eigen.hpp>

7

8 namespace stan {

9

10  namespace math {

11

20  template <typename T, typename S>

21  struct promote\_scalar\_struct<T, Eigen::Matrix<S,-1,-1> > {

22

31  static Eigen::Matrix<typename promote\_scalar\_type<T,S>::type, -1,-1>

32  apply(const Eigen::Matrix<S, -1,-1>& x) {

33  Eigen::Matrix<typename promote\_scalar\_type<T,S>::type, -1,-1>

34  y(x.rows(), x.cols());

35  for (size\_t i = 0; i < x.size(); ++i)

36  y(i) = promote\_scalar\_struct<T,S>::apply(x(i));

37  return y;

38  }

39

40  };

41

42

51  template <typename T, typename S>

52  struct promote\_scalar\_struct<T, Eigen::Matrix<S,1,-1> > {

53

62  static Eigen::Matrix<typename promote\_scalar\_type<T,S>::type, 1,-1>

63  apply(const Eigen::Matrix<S, 1,-1>& x) {

64  Eigen::Matrix<typename promote\_scalar\_type<T,S>::type, 1,-1>

65  y(x.rows(), x.cols());

66  for (size\_t i = 0; i < x.size(); ++i)

67  y(i) = promote\_scalar\_struct<T,S>::apply(x(i));

68  return y;

69  }

70

71  };

72

73

82  template <typename T, typename S>

83  struct promote\_scalar\_struct<T, Eigen::Matrix<S,-1,1> > {

84

93  static Eigen::Matrix<typename promote\_scalar\_type<T,S>::type, -1,1>

94  apply(const Eigen::Matrix<S, -1,1>& x) {

95  Eigen::Matrix<typename promote\_scalar\_type<T,S>::type, -1,1>

96  y(x.rows(), x.cols());

97  for (size\_t i = 0; i < x.size(); ++i)

98  y(i) = promote\_scalar\_struct<T,S>::apply(x(i));

99  return y;

100  }

101

102  };

103

104

105  }

106 }

107

108

109 #endif

110

111

112

113

stan::math::promote\_scalar\_struct< T, Eigen::Matrix< S,-1, 1 > >::apply

static Eigen::Matrix< typename promote\_scalar\_type< T, S >::type,-1, 1 > apply(const Eigen::Matrix< S,-1, 1 > &x)

Return the row vector consisting of the recursive promotion of the elements of the input row vector t...

**Definition:** promote\_scalar.hpp:94

promote\_scalar\_type.hpp

stan::math::promote\_scalar\_struct< T, Eigen::Matrix< S, 1,-1 > >::apply

static Eigen::Matrix< typename promote\_scalar\_type< T, S >::type, 1,-1 > apply(const Eigen::Matrix< S, 1,-1 > &x)

Return the column vector consisting of the recursive promotion of the elements of the input column ve...

**Definition:** promote\_scalar.hpp:63

stan::math::promote\_scalar\_struct

General struct to hold static function for promoting underlying scalar types.

**Definition:** promote\_scalar.hpp:21

Eigen.hpp

promote\_scalar.hpp

stan::math::promote\_scalar\_struct< T, Eigen::Matrix< S,-1,-1 > >::apply

static Eigen::Matrix< typename promote\_scalar\_type< T, S >::type,-1,-1 > apply(const Eigen::Matrix< S,-1,-1 > &x)

Return the matrix consisting of the recursive promotion of the elements of the input matrix to the sc...

**Definition:** promote\_scalar.hpp:32

stan::math::promote\_scalar\_struct::apply

static T apply(S x)

Return the value of the input argument promoted to the type specified by the template parameter...

**Definition:** promote\_scalar.hpp:34


---

[ Stan Home Page ]

*© 2011–2014,
Stan Development Team.*
